# Revisiting Glutamate Excitotoxicity in Amyotrophic Lateral Sclerosis and Age-Related Neurodegeneration

**DOI:** 10.3390/ijms25115587

**Published:** 2024-05-21

**Authors:** Frederick J. Arnold, Alexandra F. Putka, Urmimala Raychaudhuri, Solomon Hsu, Richard S. Bedlack, Craig L. Bennett, Albert R. La Spada

**Affiliations:** 1 Department of Pathology and Laboratory Medicine, University of California Irvine, Irvine, CA 92617, USA; 2 Department of Neurology, Duke University School of Medicine, Durham, NC 27710, USA; aputka@umich.edu (A.F.P.);; 3 Department of Neurology, University of California Irvine, Irvine, CA 92617, USA; 4 Department of Biological Chemistry, University of California Irvine, Irvine, CA 92617, USA; 5 Department of Neurobiology and Behavior, University of California Irvine, Irvine, CA 92697, USA; 6 UCI Center for Neurotherapeutics, University of California Irvine, Irvine, CA 92697, USA

**Keywords:** glutamate excitotoxicity, AMPA receptors, GluR2 editing, astrocytes, NMDA receptors

## Abstract

Amyotrophic lateral sclerosis (ALS) is the most common motor neuron disorder. While there are five FDA-approved drugs for treating this disease, each has only modest benefits. To design new and more effective therapies for ALS, particularly for sporadic ALS of unknown and diverse etiologies, we must identify key, convergent mechanisms of disease pathogenesis. This review focuses on the origin and effects of glutamate-mediated excitotoxicity in ALS (the cortical hyperexcitability hypothesis), in which increased glutamatergic signaling causes motor neurons to become hyperexcitable and eventually die. We characterize both primary and secondary contributions to excitotoxicity, referring to processes taking place at the synapse and within the cell, respectively. ‘*Primary pathways*’ include upregulation of calcium-permeable AMPA receptors, dysfunction of the EAAT2 astrocytic glutamate transporter, increased release of glutamate from the presynaptic terminal, and reduced inhibition by cortical interneurons—all of which have been observed in ALS patients and model systems. ‘*Secondary pathways*’ include changes to mitochondrial morphology and function, increased production of reactive oxygen species, and endoplasmic reticulum (ER) stress. By identifying key targets in the excitotoxicity cascade, we emphasize the importance of this pathway in the pathogenesis of ALS and suggest that intervening in this pathway could be effective for developing therapies for this disease.

## 1. Introduction

### 1.1. Excitotoxicity in ALS

Amyotrophic lateral sclerosis (ALS) is a progressive neuromuscular disorder characterized by the selective death of motor neurons (MNs) in the brain and spinal cord [1]. As MNs die, muscle stiffness and spasticity result, eventually leading to atrophy. ALS progresses quickly, typically resulting in death from respiratory paralysis within 3–5 years of diagnosis. Despite being the most frequent neurodegenerative disorder of midlife, FDA-approved drugs for this disease (riluzole, edaravone [1], relyvrio, and nuedexta) have only modest benefits [2,3]. Notably, the two longest-standing of these drugs, riluzole and edaravone, target processes related to the hyperexcitability of MNs [1]. *Riluzole* decreases the repetitive firing of neurons through a number of mechanisms: inhibiting voltage-gated Ca^2+^ channels to decrease neurotransmitter release, reducing Na^+^ currents to decrease depolarization of the postsynaptic neuron, and by augmenting calcium-dependent K^+^ currents to increase hyper-polarization of the presynaptic neuron [4]. Thus, riluzole decreases the excitation of lower MNs in several specific ways. *Edaravone* acts as a scavenger of free radicals to protect neurons, glia, and vascular cells from oxidative stress [5]. Oxidative stress can be caused by excitotoxicity downstream of processes at the synapse (where riluzole acts). Excess calcium influx at the postsynaptic neuron due to persistent glutamatergic signaling leads to mitochondrial dysfunction and the production of reactive oxygen species (ROS), which cause toxicity both in neurons and in surrounding astrocytes [6]. Therefore, the induction of excitotoxicity by an elevated concentration of glutamate in the synapse and its downstream effects are key drug targets, emphasizing the long-accepted importance of excitotoxicity in ALS etiology.

*Relyvrio* attained FDA approval just recently in 2022 and is composed of three parts sodium phenylbutyrate (PB) and one part taurursodiol (TURSO) (Amylyx Pharmaceuticals). Taurursodiol is thought to inhibit cell apoptosis by disrupting the mitochondrial pathway of cell death [7]. PB has been used to reduce ammonia in some urea-cycle and short-chain fatty-acid disorders. In cellular models, PB can alter transcription, reduce neuroinflammation, and improve cellular energy metabolism [8]. In humans, PB is rapidly metabolized to phenylacetate, which acetylates glutamine to form phenylacetylglutamine, a histone deacetylase inhibitor that reduces stress response signaling in the endoplasmic reticulum [9], which we will also discuss as a downstream consequence of glutamate excitotoxicity.

**Figure 1:**
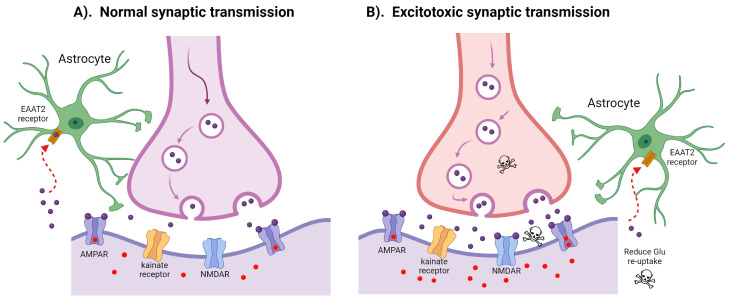
Physiologic versus excitotoxic synaptic glutamate transmission and re-uptake. (**A**) In typical synaptic transmission, physiological levels of glutamate (purple circles) are trafficked within vesicles to the synapse. Synaptic glutamate activates three ionotropic glutamate receptors expressed on the postsynaptic neuron: N-methyl-D-aspartate receptor (NMDAR), α-amino-3-hydroxy-5-methyl-4-isoxazolepropionic acid receptor (AMPAR), and kainate receptors. Regulated activation of calcium-permeable vs. calcium-impermeable AMPARs by glutamate facilitates the entry of calcium ions (red dots) into the postsynaptic cell. Balanced re-uptake of glutamate from the synapse is predominantly undertaken by EAAT2 receptors on the astrocytes (red arrow). (**B**) Prominent mechanisms underlying excitotoxic transmission include excessive glutamate release at the synapse, inefficient glutamate re-uptake via astrocytic EAAT2 receptors [10], and increased calcium permeability of AMPARs.

Motor neuron firing is a normal process that is vital for skeletal muscle function. When a wave of depolarization reaches the presynaptic terminal of upper MNs, glutamate is released into the synapse. Glutamate activates ionotropic (ligand-gated ion channels) and metabotropic receptors on the postsynaptic motor neuron, eventually causing its depolarization and firing. The critical ionotropic receptors activated by glutamate are N-methyl-D-aspartate (NMDA), α-amino-3-hydroxy-5-methyl-4-isoxazolepropionic acid (AMPA), and kainate receptors. Excessive glutamate release and overstimulation of glutamate receptors can be toxic, as they cause aberrant firing of neurons and trigger downstream pathways that lead to cell death [10]. This process is known as excitotoxicity (Figure 1).

Given that certain existing drugs approved to treat ALS likely target glutamate excitotoxicity, this process appears to play a fundamental role in ALS pathogenicity. Thus, a better understanding of how the divergent causes of ALS converge on excitotoxicity, as well as an understanding of the factors that make MNs selectively vulnerable to this threat, will meaningfully inform future therapies.

### 1.2. Excitotoxicity in Alzheimer’s Disease, Huntington’s Disease, and Parkinson’s Disease

Other neurodegenerative diseases such as Alzheimer’s disease (AD), Huntington’s disease (HD), and Parkinson’s disease (PD) have been linked to excitotoxicity through both cell-autonomous and non-cell-autonomous mechanisms [11,12]. The role of excitotoxicity in these age-related neurodegenerative disorders has been extensively reviewed [13]. In AD, amyloid-β aggregates disrupt calcium homeostasis in neurons, thereby leaving them vulnerable to NMDA and kainate receptor-mediated glutamate toxicity [14]. Similarly, in HD, injection of an NMDA receptor agonist (quinolinic acid) into the striatum of rats recapitulates the selective loss of medium spiny neurons in this disease, implicating the activation of these receptors in excitotoxicity-mediated neuronal degeneration [15]. Finally, α-synuclein aggregates, the main pathological hallmark of PD, were found to augment AMPA receptor-mediated synaptic transmission, thereby increasing cell death [16]. Increased levels of the metabotropic glutamate receptor mGluR5 have been observed in the hippocampus and basal ganglia of a transgenic (Tg) mouse model of PD, indicating that α-synuclein may directly interact with mGluR5 to cause overactivation of this receptor, leading to excitotoxic cell death [17].

Non-cell-autonomous mechanisms also contribute to excitotoxicity in these prominent neurodegenerative disorders, as decreased expression of the astrocytic glutamate transporter EAAT2 (also known as SLC1A2) has been reported in AD, HD, and PD [18,19,20]. Microglia are also implicated in disease. Wildtype rat microglia activated by pro-inflammatory stimuli, namely lipopolysaccharide (LPS), release glutamate, causing neuronal damage [21]. Increased glutamate levels affect the Xc exchange system, essential for glutathione synthesis, as well as NADPH oxidase activation in LPS-triggered microglia. The activation of oxidative processes downstream of glutamate release is a key topic of excitotoxicity which we will later address. Additionally, glutamate can activate microglia in rat brains via metabotropic glutamate, AMPA, and kainate receptors, leading to the release of pro-inflammatory cytokines and exacerbating inflammation [22]. The contribution of oligodendrocytes, the final glial cell in the central nervous system, to motor neuron excitotoxicity remains relatively unexplored. However, there is increased evidence of oligodendrocyte dysfunction in ALS [23,24,25], AD [26], HD [27,28], PD [29], and many other neurodegenerative disorders [30]. Given that oligodendrocytes are essential to long-term axonal integrity and neuron health [31], the contribution of oligodendrocytes to excitotoxic cell death remains a pressing area of future research. In sum, excitotoxicity is a common pathogenic mechanism of neurodegenerative disease; however, in each disease, there are unique upstream molecular and cellular processes that converge upon excitotoxicity.

### 1.3. Excitotoxicity Pathways as Druggable Targets

Given the evidence of excitotoxicity in neurodegenerative diseases, therapies that target this mechanism would seem broadly applicable. Riluzole was deemed effective in treating a rat model of PD [32] and a mouse model of HD [33], but was not effective in human HD [34] and PD patients [35], both representing failed clinical trials. Two Phase II trials (NCT03605667 and NCT01703117) testing the use of riluzole in a pro-drug format (troriluzole) for the treatment of AD are currently underway, and the results are not yet available [36,37]. Edavarone has shown promise as a neuroprotective agent in in vitro and in vivo models of AD, HD, and PD. It was shown to lower amyloid-β plaque levels in APP/PS1 Tg mice, which recapitulate a few key features of AD [38]. Edaravone treatment improved the phenotype of male Wistar rats injected with quinolinic acid to mimic early-stage HD [33], as well as mouse neuroblastoma N2a cells transfected with *DJ-1* (PARK7) containing the L166P mutation, which has been linked to early-onset familial PD [39]. However, the efficacy of edaravone in human patients remains to be determined. While riluzole and edaravone have clearly not emerged as effective treatments for neurodegenerative disease, excitotoxicity is underscored as a convergent disease mechanism in ALS, AD, HD, and PD. Thus, excitotoxicity could be a drug target common to these diseases, if appropriate human-specific targets emerge.

### 1.4. Primary and Secondary Downstream Pathways of Excitotoxicity

Here, we highlight the existing evidence for the role of excitotoxicity in ALS disease pathogenesis. We classify the factors contributing to excitotoxicity as primary (upstream) or secondary (downstream) processes. We include examples of cell-autonomous and non-cell-autonomous contribution to excitotoxicity within each category, noting that they are not mutually exclusive. We define primary processes as those that occur at the synapse, including synaptic accumulation of glutamate and the resulting postsynaptic influx of calcium ions. We also discuss the effect of inhibitory interneurons on excitatory transmission. Secondary processes include the neural response to elevated intracellular calcium levels by calcium-buffering organelles such as mitochondria and the ER. Importantly, many of these secondary processes are intimately connected to ALS pathobiology (e.g., mitochondrial dyshomeostasis and activation of the unfolded protein response (UPR)).

For the treatment of sporadic ALS (SALS), which accounts for the vast majority of ALS cases, future therapies must target convergent mechanisms of disease. Here, we discuss primary and secondary processes that all converge on excitotoxicity in ALS, highlighting potential targets for biomarkers and therapeutics.

## 2. Primary Excitotoxic Processes

Excitotoxicity can be caused by an increase in the sensitivity of the postsynaptic neuron to normal levels of glutamate (referred to as slow excitotoxicity), an increase in the concentration of glutamate in the synapse (referred to as classical excitotoxicity), or both [2,10].

### 2.1. AMPA Receptor Subunit Composition and GluR2 Editing

**Figure 2:**
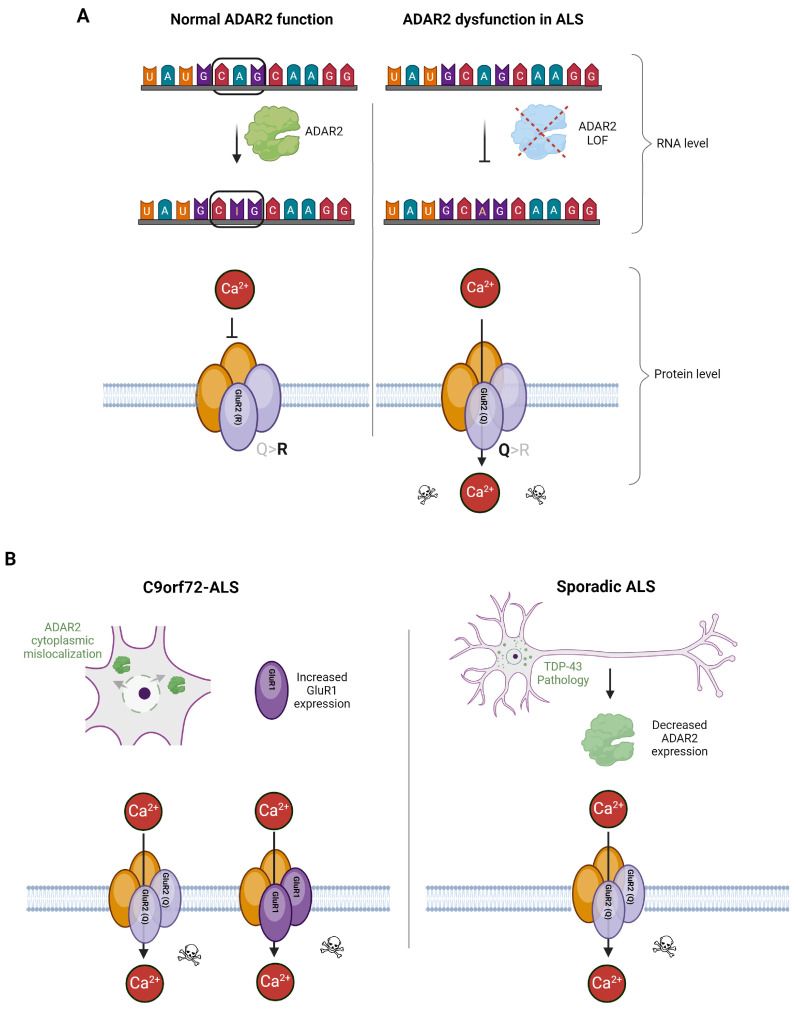
GluR2 editing by the ADAR2 enzyme and AMPAR composition in ALS. (**A**) The ADAR2 enzyme converts the GluR2 mRNA CAG codon to a CIG codon at the RNA level (**left panel**). This A-to-I RNA editing changes the gene-encoded glutamine to a positively charged arginine residue at the protein level. The incorporation of ADAR2-edited GluR2 results in a calcium-impermeable AMPAR (**left panel**). This RNA editing dysfunction allows calcium permeability of the AMPAR (right panel) and downstream excitotoxicity [10]. (**B**) In both FALS and SALS, multiple mechanisms have been proposed that ultimately result in increased calcium permeability of AMPARs. In C9orf72-ALS (**left panel**), nucleocytoplasmic mislocalization of ADAR2 has been observed in multiple model systems as well as in postmortem patient spinal cord tissue. As described, this leads to reduced editing of the GluR2 mRNA and an increase in AMPAR calcium permeability [44]. Additionally, increased expression of calcium-permeable GluR1 was reported in C9orf72-ALS iPSC-derived motor neurons. In SALS (**right panel**), TDP-43 pathology corresponds with decreased ADAR2 expression, which also results in decreased editing of the GluR2 mRNA and increased calcium permeability via AMPARs.

Excitatory transmission is regulated by glutamate-mediated activation of the ionotropic NMDA receptors (NMDARs) and AMPA receptors (AMPARs). AMPA receptors are tetramers formed from a varying combination of four subunits: GluR1, GluR2, GluR3, and GluR4 [10]. Both NMDARs and AMPARs are permeable to sodium and potassium ions, but NMDARs are also permeable to calcium. The subunit composition of AMPARs determines the calcium permeability of the receptor. Specifically, receptors containing the GluR2 subunit are impermeable to calcium, while those lacking the GluR2 subunit are calcium-permeable [10] (Figure 2).

GluR2-containing AMPARs are impermeable to calcium due to the presence of a positively charged arginine residue at position 586 [10,40,41,42]. Transcripts encoding R586 are generated when the RNA editing enzyme ADAR2 converts the genetically encoded adenine to inosine in the GRIA2 pre-mRNA (henceforth referred to as GluR2 mRNA). This creates a CIG triplet, where the inosine is read as guanosine to encode an arginine at position 586. This editing of a glutamine to an arginine (Q/R editing) makes GluR2-containing receptors impermeable to calcium and is effectively complete in most cells; however, in ALS, multiple studies have observed decreased ADAR2 editing of GluR2 [10,43]. These unedited subunits increase the calcium uptake of ALS MNs and represent a potential therapeutic target in ALS [44].

The efficiency of GluR2 editing is significantly reduced in the ventral horn gray matter of ALS patients compared with healthy controls or patients with non-ALS neurological disorders [45]. Additionally, lowered Q/R editing efficiency in ALS corresponds with lower expression of GluR2 mRNA in the same regions [45]. Supporting this point, in a study of RNA extracted from single MNs, it was discovered that GluR2 editing was incomplete in 56% of ALS cases, while 100% of controls exhibited complete editing [46]. Thus, the etiology of ALS is closely linked to lowered Q/R editing and lowered GluR2 mRNA expression, both of which lead to increased calcium influx due to the presence of calcium-permeable AMPARs in the cell membrane (Figure 2B).

Importantly, reduced Q/R editing of GluR2 is sufficient to cause neuromuscular disease in mice. Mice lacking ADAR2 function (ADAR2^−/−^) die between P0 and P20 and exhibit seizures after P12, likely due to the 30-fold higher Ca^2+^ permeability of ADAR2^−/−^ neurons compared to controls [47]. This study revealed the importance of ADAR2 function in early development. Moving beyond the first weeks of life, a conditional knock-out (KO) of the ADAR2 gene resulted in reduced motor function and increased motor neuron death [48]. By 5 weeks of age, roughly 50% of MNs lacked ADAR2 activity. Neuronal loss was selectively observed in the spinal cord and nuclei of cranial motor nerves [48]. Intriguingly, oculomotor neurons, which are typically spared in ALS, were resistant to cell death despite a significant decrease in GluR2 editing [48]. Another group generated a Tg mouse model by changing the GluR2 sequence to encode asparagine (GluR2-N) at the Q/R site, which makes editing impossible [49]. Heterozygous expression of the GluR2-N transgene resulted in a two-fold increase in calcium permeability of AMPARs in the Tg mice [49], reviewed in [10]. These mice developed motor neuron degeneration later in life. The ADAR2 conditional KO mice noted above were recently employed with selective ADAR2-KO in cholinergic neurons. This resulted in a significant reduction in MNs in the lateral anterior horn and an increase in reactive astrocytes [50]. Conversely, when endogenous GluR2 alleles in ADAR2 KO mice were mutated to an unchangeable arginine residue, there was no neurodegeneration and mice had normal motor function [48]. Therefore, selective loss of ADAR2 in MNs is sufficient to produce an ALS-like phenotype in vivo, while ensuring arginine inclusion in GluR2 ameliorates features of disease.

Understanding the factors contributing to the loss of ADAR2 in ALS patients requires investigating various genetic models of disease, including TAR DNA-binding protein-43 (TDP-43) and C9orf72. TDP-43 is a heterogeneous nuclear ribonucleoprotein (hnRNP) that binds to RNA as well as DNA [51]. The hnRNP protein family plays numerous roles in mRNA and pre-mRNA processing, regulating gene splicing, stability, export, and translation [51]. In postmortem brain samples from about 97% of ALS patients, TDP-43 is found in ubiquitin-positive cytoplasmic inclusions that also exhibit abnormal phosphorylation and TDP-43 nuclear clearance [52]. Indeed, the presence of TDP-43 cytoplasmic accumulations in the central nervous system (CNS) is the most common pathological hallmark of ALS. The most notable exception is familial ALS (FALS) patients with the superoxide dismutase-1 (SOD1) mutation, who instead display inclusions of SOD1 itself [52].

In spinal cord tissue from SALS patients, TDP-43 pathology in MNs correlated with reduced expression of ADAR2 [53], suggesting a link between TDP-43 and ADAR2. ADAR2 was found to be upregulated during mouse development, and its mRNA expression decreased progressively with age in the spinal cord [54]. In control mice, MNs typically exhibit nuclear TDP-43 and ADAR2 localization, but in aged mice particularly, fast fatigable MNs show reduced ADAR2 nuclear staining and TDP-43 cytoplasmic redistribution. These particular MNs also showed evidence of the unedited GluR2 mRNA, suggesting that such age-related changes may represent risk factors for ALS [54]. Using Neuro2a cells, ADAR2 activity was unchanged in response to knockdown, overexpression, mutant expression, or cleavage fragments of TDP-43. This suggests that TDP-43 perturbation is not an upstream event of GluR2 editing defects per se [55].

While reduced ADAR2 expression has been reported in SALS, ADAR2 function may be altered via nucleocytoplasmic mislocalization in C9orf72 ALS/frontotemporal dementia (FTD) [56]. C9orf72-ALS, caused by a GGGGCC (G4C2) hexanucleotide repeat expansion in the first intron of the *C9ORF72* gene, is the most common genetic cause of ALS, accounting for approximately 40% of familial cases and 8% of sporadic cases [57,58]. The *C9ORF72* gene is bidirectionally transcribed, and its unspliced, repetitive intronic RNAs are translated in all possible reading frames to yield five unique dipeptide repeat proteins (DPRs) via repeat-associated non-ATG (RAN) translation [57]. Motor neuron death in C9orf72-ALS is likely caused by the combined effects of (i) haploinsufficiency of the C9orf72 protein, (ii) gain of toxic function caused by the accumulation of G4C2 RNA into nuclear foci, and (iii) by RAN translation of G4C2 repeat RNA into aberrant DPRs [57,59,60]. In C9orf72 ALS/FTD, ADAR2 was found to be mislocalized in postmortem spinal cord tissue from C9orf72 patients, hiPSC MNs derived from C9orf72 patients, and in 6-month-old mice expressing the hexanucleotide repeat expansion via P0 intracerebroventricular (ICV) injection with AAV-(G4C2)149 [56]. This is in contrast to SALS cases, in which ADAR2 was downregulated rather than mislocalized in the MNs [48]. Furthermore, RNA sequencing of postmortem CNS tissue from patients with C9orf72 ALS/FTD revealed hypo-editing of GluR2 in the motor cortex and hyper-editing of GluR2 in the spinal cord [56]. In contrast, another study found increased expression of the calcium-permeable GluR1 AMPAR subunit in iPSC-derived MNs from ALS patients with the *C9ORF72* mutation [61]. Since they observed complete editing of the GluR2 subunit in motor neuron culture, Selvaraj et al. attributed the rise in the calcium permeability of AMPARs to the increased expression of the GluR1 subunit. Altogether, while it is unclear precisely how the *C9ORF72* mutation affects GluR2 editing, there is evidence that it leads to changes in AMPAR composition and calcium permeability. Moreover, because postmortem tissues were used in the study by Moore et al. (2019) [56], it is unknown if the downregulation of GluR2 that they observed is a facet of the early stage or late stage of disease.

In both SALS and FALS, there is evidence for loss of ADAR2 function resulting in unedited GluR2. This increases the calcium permeability of AMPARs, thus contributing to excitotoxicity. MNs are especially susceptible to increased intracellular calcium levels due to low expression of Ca^2+^-buffering proteins, resulting in a poor capacity for Ca^2+^ buffering [10]. This necessarily increases the role that calcium-buffering organelles such as mitochondria and the ER must play to protect the cell from excess calcium (as will be discussed below).

### 2.2. Non-Cell-Autonomous Contribution: Astrocytic Glutamate Transporters

In addition to cell-autonomous regulation of the glutamate response by AMPAR subunit composition, non-cell-autonomous regulation by glia is also critically important. Under normal conditions, the extracellular concentration of glutamate is highly regulated by astrocytic uptake from the synapse [2]. Evidence exists that this process is disrupted in ALS [62] due to increased glutamate release from the presynaptic terminal and/or by disruption of glutamate transporters. Ultimately, the presence of excess glutamate in the synapse leads to excitotoxicity via chronic stimulation of the postsynaptic neuron.

The EAAT family of glutamate transporter proteins 1–5 control the re-uptake of glutamate from the synapse. The members of this protein family are differentially expressed in the cells of the brain. EAAT3–EAAT5 are expressed throughout the brain, with EAAT4 and EAAT5 enriched in Purkinje cells in the cerebellum and in retinal photoreceptors and bipolar cells, respectively [62]. Of interest here, EAAT1 and EAAT2 are primarily expressed in astrocytes, though they are also expressed by other glial cells [63,64]. Specifically, EAAT1 is expressed by astrocytes in the cerebellum, while EAAT2 is highly expressed by astrocytes throughout the CNS. Since EAAT2 is highly abundant in the brain, comprising up to 1% of total brain protein [64], it is thought to be the primary transporter responsible for glutamate clearance from the synapse. While neurons also express EAAT transporters to re-uptake glutamate, these transporters are less abundant than the transporters expressed by astrocytes [62,65]. Altered expression of glutamate transporters is a feature of several neurodegenerative disorders, including PD, HD, and ALS [62,66]. Early studies suggested that loss of EAAT2 was sufficient to induce motor neuron degeneration in mice [67]. Additionally, knockdown of EAAT2 in adult rats by antisense oligonucleotides led to progressive motor neuron degeneration [63]. These studies suggest that EAAT2 is important to the health of MNs.

In SALS, up to 60–70% of patients have a 30–95% loss of EAAT2 protein in the motor cortex and spinal cord [68]. This may be caused by aberrant processing of the *EAAT2* mRNA, as multiple truncated forms of *EAAT2* were found in the motor cortex (but not the cerebellum or hippocampus) of ALS patients [68]. Thus, unaffected areas had normal *EAAT2* mRNA, while affected areas had aberrantly spliced *EAAT2* mRNA species, including variants with retained introns and alternative exon skipping. These aberrant *EAAT2* mRNAs were found in 65% of SALS patient samples and corresponded with a loss of EAAT2 protein in vivo, as proteins translated from abnormal *EAAT2* isoforms are unstable and rapidly degraded. Mis-spliced *EAAT2* mRNAs were found in the CSF of living ALS patients, indicating that these mRNAs are expressed early in disease.

Weakening the hypothesis that aberrant *EAAT2* mRNAs are the cause of EAAT2 loss in SALS, alternatively spliced *EAAT2* transcripts have been found in regions of the CNS unaffected by disease in patients with ALS, and in control subjects [69,70]. Thus, the causes of *EAAT2* downregulation or loss are not fully understood. In addition to the uncertain correlation between alternative splicing of *EAAT2* and overall EAAT2 levels, both oxidative and nitrosative stressors have been shown to inactivate transporter activity [71]. This suggests a potential feedback mechanism in which excitotoxicity in ALS can further decrease EAAT2 activity by increasing cellular oxidative stress [72] (as will be discussed). Altogether, the prevalence of mis-spliced *EAAT2* mRNAs seems to play a factor in SALS patients, but its exact role is unclear. Additionally, FALS mutations have also been linked to loss of EAAT2. Expression of mutant SOD1 leads to oxidative damage of the EAAT2 protein [73], which provides another possible mechanism underlying EAAT2 downregulation. Likewise, treatment of human astrocytes with exogenous DPRs led to mis-splicing of *EAAT2* [74], recapitulating what was previously observed in ALS patient tissue [68]; however, a separate study found that DPRs directly bind to the EAAT2 mRNA but were unable to reproduce the splicing defect [75]. Loss of C9orf72 also leads to a 1.9-fold and 2.5-fold decrease in EAAT2 receptors in U87 glioblastoma cells and the human prefrontal cortex, respectively [71]. This correlates with the accumulation of glutamate upon knockdown of C9orf72 in cells.

**Figure 3:**
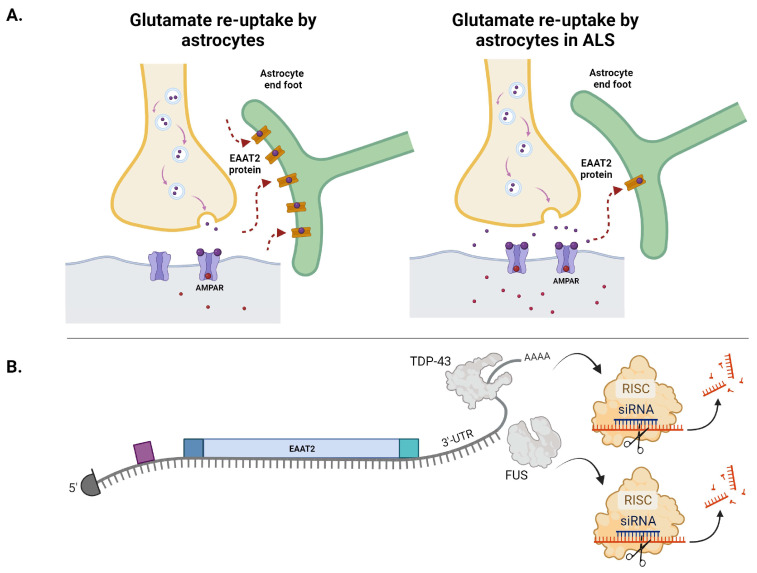
EAAT2 re-uptake of synaptic glutamate and EAAT2 mRNA dysregulation in ALS. (**A**) Astrocytes play an important role in the re-uptake of excess glutamate from the synaptic cleft. While the precise etiologic mechanism remains unknown, EAAT2 protein levels are significantly reduced in the motor cortex and spinal cord of ALS patients [68]. Such EAAT2 protein loss has been suggested to result from factors such as oxidative stressors, aberrant splicing mechanisms, and toxic DPRs generated from the C9orf72 expansion in FALS. (**B**) The ALS-associated RNA binding proteins (RBPs) TDP-43 and FUS are known to bind within the 3′UTR of EAAT2 mRNA [79]. Altered RBP interactions with the EAAT2 3′UTR may change cis-regulatory element regulation of the EAAT2 transcript, such as increased miRNA binding, as one possible mechanism contributing to EAAT2 loss in ALS.

In *Drosophila*, TDP-43 overexpression upregulates and knockdown downregulates mRNA levels of EAAT1 and EAAT2, respectively [76]. This suggests that these transporters are directly regulated by TDP-43, and indeed, TDP-43 binds to the 3′UTR region of *EAAT2* mRNA [77] (Figure 3). ALS-associated mutations in the DNA/RNA binding protein fused in sarcoma (FUS) have also been linked to *EAAT2* mis-splicing and dysregulation, as FUS also directly binds within the 3′UTR of *EAAT2* [78] (reviewed in [79]) (Figure 3B).

More work is required to fully elucidate the temporal nature of EAAT2 downregulation, which may occur as a consequence of neuron loss or may precede (and contribute to) motor neuron loss [68]. SOD1-G93A Tg mice exhibit no changes in EAAT2 levels at 14 weeks old [80]; however, decreased immunoreactivity of EAAT2 was found in the ventral horn of the lumbar spinal cord in 14- to 18-week-old mice, which suggests downregulation prior to symptoms in SOD1 ALS. Altogether, while there are multiple connections between the proteins linked to FALS and EAAT2 function, the mechanism of such alterations is not clear in all cases. Notably, because TDP-43 pathology is a feature of SALS, EAAT2 regulation by TDP-43 may be disrupted in the vast majority of ALS patients during the disease course.

Determining whether EAAT2 is lost early or late in the different forms of ALS disease progression could have important therapeutic implications, as downregulation of EAAT2 early in ALS could make it a useful biomarker. Current biomarkers of ALS include measuring cortical hyperexcitability through transcranial magnetic stimulation or electromyography, which may indicate overall EAAT2 transporter activity [81]. There is already some precedent for targeting the EAAT2 transporter as a therapy in ALS, as riluzole upregulates EAAT2 activity in spinal cord synaptosomes from rats [82].

### 2.3. Increase in Presynaptic Glutamate Release

Aside from a decrease in astrocytic EAAT2 transporters, there could be a greater concentration of glutamate in the synapse due to increased glutamate release from the presynaptic neuron. Neurotransmitters are released into the synapse when a depolarizing wave reaches the presynaptic terminal, which opens voltage-gated calcium channels. Calcium enters the cell and catalyzes the fusion of synaptic vesicles with the presynaptic membrane, releasing neurotransmitters into the synapse. Therefore, increased release of glutamate could be due to hyperactivity of the presynaptic neuron (related to depolarization) and/or calcium dysregulation at the terminal [2].

Most of our understanding of increased glutamate release in ALS comes from the mutant SOD1 G93A Tg mouse model. Transporters for different neurotransmitters are found in the same bouton of a neuron, meaning there are mixed populations of homotransporters and heterotransporters [83]. Homotransporters re-uptake the neurotransmitter that was just released into the synapse, while heterotransporters re-uptake neurotransmitters released from neighboring neurons. Activation of either glycine or GABA heterotransporters at glutamatergic terminals can cause greater release of glutamate in mice expressing mutant human SOD1 compared to mice expressing wildtype human SOD1 [83]. These findings suggest that mutant SOD1 exhibits gain of function in relation to glutamatergic neurotransmission, as evidenced by enhanced glutamate release in this model of FALS [83].

Another study using the same SOD1 G93A Tg mouse model found increased calcium concentration in synaptosomes under resting and depolarizing conditions [84]. Calcium-modulating kinase protein II (CaMKII) phosphorylates synapsin I, which leads to the mobilization of vesicles from the reserved pool to the readily releasable pool [84]. Synapsin I phosphorylation is increased in SOD1 G93A mice, suggesting that these mice have a larger readily releasable pool of glutamate-containing synaptic vesicles than control mice. The findings of Milanese et al. (2011) [84] thus suggest a mechanism for the increased synaptic glutamate concentration observed in the SOD1 G93A model by Raiteri et al. (2004) [83]. While these results suggest a possible mechanism for increased synaptic glutamate and excitotoxicity, we must also acknowledge that only 10–20% of patients with FALS harbor SOD1 mutations [85], and SOD1 ALS represents a major outlier in that the hallmark ALS pathology of TDP-43 aggregates are absent in this form of disease [1]. Hence, these hypotheses should be tested in other models of FALS, as well as models of SALS (once available) [86], to provide a more complete mechanistic understanding.

Thus far, we have discussed how three processes occurring at an excitatory synapse may contribute to excitotoxicity: (i) decreased editing of the GluR2 subunit causes greater calcium influx into postsynaptic MNs via calcium-permeable AMPA receptors; (ii) decreased expression of the astrocytic glutamate transporter EAAT2 reduces the re-uptake of glutamate from the synapse; and (iii) increased calcium in the presynaptic terminal of SOD1-G93A mice can lead to a larger pool of synaptic vesicles containing glutamate. These latter two processes both contribute to an increased synaptic concentration of glutamate. However, to complete the picture of synaptic contributions to excitotoxicity, we must investigate the role of inhibitory neurons that synapse onto MNs.

### 2.4. Inhibitory Neuron Contributions

Decreased inhibition of MNs due to impaired inhibitory inputs could lead to the observed hyperexcitability of MNs in ALS; however, there are conflicting ideas on the role that inhibitory neurons play in ALS pathogenesis. Using hSOD1-G93A mice to study excitability across the lifespan, one study found an interesting pattern that suggested homeostatic modulation of cortical excitability. Several days after birth (P5–P6), mutant SOD1 mice exhibited changes in excitability measured through patch-clamp recordings, but in juvenile, pre-symptomatic mice (P26–P40), the properties of cortical neurons were normal [87]. It was not until symptomatic disease (P90–P129) that the cortical neurons became hyperexcitable again. Yet at this same time point (P90–P129), no significant differences were observed in corticospinal neuron frequency of activity between hSOD1-G93A versus control mice [87]. This suggests that even in late-stage disease, other cells in the circuit may work to counterbalance the abnormal hyperexcitability of corticospinal neurons, meaning that homeostatic plasticity acts on a broad level to prevent overabundant excitation of a circuit. Thus, it seems that ALS onset alters cortical neuron subtypes that have unique cellular responses during the disease course.

Inhibitory interneurons also play a role, as parvalbumin (PV) interneurons exhibit increased intrinsic excitability in hSOD1-G93A ALS mice. This coincides with progressive decreases in dendritic arbor size and spine density in pyramidal neurons of the motor cortex of hSOD1-G93A mice [88,89], suggesting that an increase in PV interneuron excitability during the late stages of disease could arise out of the need to compensate for altered inputs in the motor cortex. This may explain why symptomatic hSOD1-G93A mice have similar corticospinal neuron activity in vivo to that of control mice. Another study confirmed altered excitability of cultured cortical interneurons in hSOD1-G93A mice, finding a decrease in the excitability of these neurons [90]. Furthermore, the authors showed that increased excitability in cortical pyramidal neurons changes the morphology of cortical interneurons; namely, that the arbor of such neurons can undergo activity-dependent remodeling. Thus, they postulate that early in disease, hSOD1-G93A drives an imbalance of excitation and inhibition in developing networks. This suggests that both excitatory and inhibitory cortical neurons experience functional abnormalities in neuron cultures from the SOD1 mouse model, emphasizing the importance of plasticity in regulating the overall excitability of neuronal circuits.

While corticospinal neurons were not hyperexcitable in symptomatic mice, they did exhibit elevated intracellular calcium levels [90]. Perhaps elevated calcium levels induce cellular dysfunction and aberrant neurotransmitter release that can initially be compensated for, but later in disease, as this positive feedback cycle continues, plasticity fails to control the downstream effects of increased calcium. In sum, these studies suggest the existence of synaptic mechanisms that maintain homeostasis across the course of the disease, keeping cortical activity in check. There remains a need to investigate exactly how such an imbalance of excitability arises, as there are many possible permutations of events. It would be useful to know what factors initially affect interneuron morphology and whether excitotoxic dysregulation precedes or succeeds interneuron dysfunction in ALS. Finally, we need to understand which model systems (if any) can be used to faithfully recapitulate the complex human circuit.

Using a TDP-43^A315T^ Tg mouse model of ALS, another group investigated cortical inhibition by recording miniature inhibitory postsynaptic currents (mIPSCs) and evoked IPSCS (eIPSCs) from layer-5 pyramidal neurons in the motor cortex of 30-week-old mice [91]. Both forms of IPSCs were significantly reduced in TDP-43 mice compared to WT littermates. Moreover, GABAergic synapse densities of layer-5 pyramidal neurons (L5-PNs) were also decreased in diseased mice. This indicates a defect in cortical inhibition in TDP-43 mice, perhaps causing pyramidal neurons to become hyperexcitable. Cellular changes were also assessed across the timeline of disease progression. At an early stage (9 weeks), there were appearances of dendritic blebs, an early sign of excitotoxicity. In late disease (15 weeks), ubiquitin-positive aggregates were observed along with a significant decrease in the number of pyramidal neurons. Altogether, these results suggest that impaired inhibitory transmission (observed at 3 weeks) leads to excitotoxicity (signs observed at 9 weeks) and neurodegeneration (observed at 15 weeks) of pyramidal neurons in TDP-43 mice. This provides a possible timeline for the sequential order of events in TDP-43 ALS.

Having established when GABAergic transmission becomes impaired, the authors next investigated where this impairment originates in the brain by measuring the activity of PV-expressing interneurons along with somatostatin (SST)-expressing interneurons, which disinhibit layer-4 pyramidal cells in the somatosensory cortex [92,93]. Using whole-field optogenetic stimulation, activation of SST interneurons was shown to inhibit PV interneurons, causing disinhibition (and thus hyperexcitability) of pyramidal neurons [91]. At 3 weeks, the same time point at which IPSCs were reduced in TDP-43 mice, SST interneurons were indeed hyperactive and PV interneurons were hypoactive [91]. The hyperactivity of SST interneurons persisted into adulthood, and these neurons also increased in number. In contrast, PV interneurons were normal in adult mice, indicating that the hyperactivity seen in young mice did not persist with age.

To further test if excitotoxicity of L5-PNs is caused by hyperactive SST interneurons, Zhang et al. (2016) [91] selectively expressed diphtheria toxin receptor (DTR) in SST neurons and then injected diphtheria toxin (DT) in 6-week-old mice, ablating these neurons. Theoretically, this could be protective, in that this would relieve the inhibition of PV interneurons and allow these neurons to keep the excitability of pyramidal neurons in check. Indeed, in 8-week-old mice (2 weeks after DT administration), the spiking activity of layer-5 pyramidal neurons matched those of control animals, indicating a lack of hyperexcitability. Moreover, in 12-week-old mice (6 weeks after DT administration), they reported (i) significant increases in GABAergic synaptic density on L5-PNs, (ii) reduced ubiquitin-positive aggregates in the primary motor cortex, and (iii) no loss of neurons in the primary motor cortex. Therefore, ablating SST neurons rescued the cellular abnormalities previously described in 9-week-old and 15-week-old TDP-43 mice. While this provides a novel approach for ameliorating the hyperexcitability of L5-PNs, it also demonstrates the importance of studying neural networks in ALS models. Looking at the entire circuit provides a more comprehensive understanding of how excitotoxicity develops, emphasizing that it is not solely due to dysregulation of excitatory neurons, but that inhibitory neurons may also play an important role. In summary, many possible mechanisms contribute to the aberrant excitability of MNs, including changes in interneuron circuits and homeostatic plasticity. Future work should elucidate the connection between different interneuron populations and MNs, as well as how dysregulation of excitability corresponds to disease stages.

## 3. Secondary Excitotoxic Processes

We will now describe ‘secondary’ excitotoxic pathways, which is how proteins and organelles respond to the increase in calcium that is characteristic of excitotoxicity. It is crucial to keep in mind that our suggested categories of primary and secondary pathways of ALS do not exist in isolation. Disease pathogenesis likely operates in feedback loops that include biological processes in each of these two pathways. As we will discuss in this section, ER and mitochondrial calcium buffering greatly influence how the cell responds to increased intracellular calcium entering through calcium-permeable AMPARs.

### 3.1. Mitochondrial Contribution

**Figure 4:**
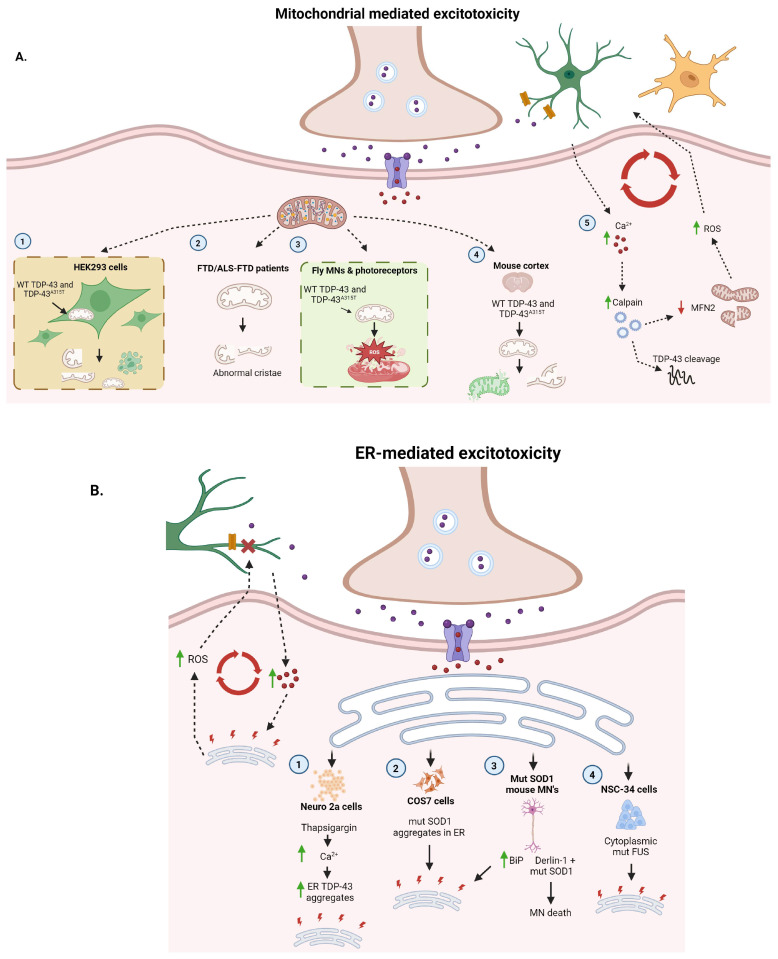
Mitochondrial and ER involvement in secondary excitotoxicity processes. (**A**) (1) In HEK293 cells, mitochondrial localization of WT TDP-43 or TDP-43^A315T^ inhibits mitochondrial complex 1, resulting in reduced mitochondrial size, abnormal/loss of cristae, and cell death. (2) Electron microscopy of mitochondria within multiple ALS/FTLD-TDP brain regions shows altered mitochondrial morphology, such as abnormal or loss of cristae. (3) In Drosophila photoreceptors, transgenic expression of WT TDP-43 and TDP-43^A315T^ alters mitochondrial morphology and increases intracellular ROS. (4) Similarly, Lentiviral overexpression of WT TDP-43 or TDP-43^A315T^ in mouse cortices leads to fragmentation and dysfunction of mitochondria and cell death. (5) Through various mechanisms discussed here, excess synaptic glutamate leads to increased intracellular calcium in MNs which are sensitive to calcium dysregulation. This activates calpain which cleaves TDP-43 and generates aggregation-prone TDP-43 fragments. Calpain also degrades MFN2, leading to impaired mitochondrial function, induction of cell death, and increased ROS. Elevated ROS can disrupt astrocyte (green) and microglia (yellow) function, further contributing to elevated synaptic glutamate and intracellular calcium. (**B**) Proteins implicated in ALS, such as SOD1, TDP-43, and FUS, are all linked to ER stress and UPR induction. (1) Treating Neuro2a cells with Thapsigargin, which increases intracellular Ca^2+^, causes cytoplasmic TDP-43 aggregation, including C-terminal TDP-43 fragments that colocalize with the ER. (2) Cos7 cells transfected with mutant (mut) SOD1 (but not WT) exhibit SOD1 aggregates localized to the ER and increased ER stress. (3) Similarly, spinal cord MNs of mutant SOD1 transgenic mice display an increase in ER chaperone proteins, such as BiP, indicative of ER stress. Furthermore, an interaction between SOD1 and Derlin-1, a member of the ER-associated degradation (ERAD) machinery, induces ER stress via activation of ASK1, resulting in MN death. (4) Like TDP-43, mutant FUS exhibits nuclear clearance and cytoplasmic aggregation in ALS. In mouse NSC-34 cells, redistribution of mutant FUS to the cytoplasm triggers ER stress. Together, these pathways support a feedback loop that can be initiated via either increased intracellular Ca^2+^ or ER stress.

Mitochondrial dysfunction in ALS involves defective oxidative phosphorylation, impaired calcium-buffering capacity, impairment of mitochondrial dynamics, and the production of reactive oxygen species (ROS) [94]. ROS production is dangerous, as it can destroy membrane integrity through multiple mechanisms, including damaging lipid components of the membrane and inducing leakiness of ion channels [95] (Figure 4A). In addition to causing damage in neurons, ROS can escape MNs to disrupt glutamate transporters on nearby astrocytes [6]. Notably, early clinical trials targeting ROS and/or mitochondria dysfunction were disappointing, suggesting that this may not be a primary driver of ALS [94]. Such studies include those of Coenzyme Q10 (NCT00243932), Dexpramipexole (NCT01281189), and Creatine [96,97,98]. Furthermore, Olexisome (NCT00868166) did not have a significant benefit in ALS patients already being treated with riluzole [99]. Interestingly, edaravone also targets ROS by acting as a free radical scavenger, and it may have a small beneficial effect on human ALS progression [100]. It is unclear why this drug that targets ROS modestly extended patient lifespan.

Several genes implicated in FALS may affect mitochondrial homeostasis via mitophagy [101]. For example, mutant SOD1 is localized to mitochondria and can interfere with mitochondrial function [10]. Several studies have demonstrated that TDP-43 can localize to mitochondria and cause mitochondrial dysfunction in TDP-43 animal and cell models [102,103]. One study analyzed electron microscopy images of patient samples to study mitochondrial morphology corresponding with mitochondrial defects [104]. These patients had a diagnosis of either frontal–temporal lobe dementia (FTLD)-TDP or ALS-FTLD-TDP, meaning they all exhibited TDP-43 pathology. The authors found notable signs of mitochondrial impairment, such as abnormal cristae or loss of cristae. The study also used cellular and animal models of TDP-43 to investigate other changes in mitochondrial function. In HEK293 cells, overexpressed mutant TDP-43^A315T^ localized to the mitochondria and corresponded with reductions in mitochondrial size as well as impaired cristae formation. Additionally, TDP-43^A315T^ induced cell death by suppressing the activity of mitochondrial complex I, which is involved in ATP synthesis. Similar evidence of mitochondrial impairment was found in vivo, as transgenic expression of both WT TDP-43 and TDP-43^A315T^ decreased mitochondrial size and increased the number of swollen or vesicular cristae in *Drosophila* photoreceptors [104]. Another study investigated the hypothesis that TDP-43 mitochondrial localization is a requirement for toxicity [105]. Lentiviral overexpression of WT TDP-43 or TDP-43^A315T^ in mouse cortices led to fragmentation and dysfunction of mitochondria, eventually contributing to cell death. These changes to mitochondrial morphology and function were abolished when TDP-43 entry to mitochondria was suppressed via deletion of the specific internal hydrophobic residues within TDP-43 required for mitochondrial localization. Compared to WT TDP-43 expression, greater mitochondrial fragmentation and cell death were observed upon mutant TDP-43 expression, and these changes were also blocked by limiting TDP-43 mitochondrial localization with targeting peptides [105]. In Drosophila models of ALS, mitochondria were shown to be a major source of ROS upon transgenic expression of WT or TDP-43^A315T^, specifically in MNs. In each case, the authors found significantly higher levels of mitochondrial ROS in Tg flies compared to non-Tg control flies [104].

As we discussed previously, activation of ionotropic glutamate receptors allows calcium to flow into the cell, and under conditions of excitotoxicity, intracellular calcium levels are increased due to excess glutamate in the synapse [11]. MNs are especially sensitive to calcium dysregulation due to several intrinsic properties. First, the expression of calcium-permeable AMPARs allows more calcium to enter the cell. Second, MNs exhibit low expression of calcium-buffering proteins such as parvalbumin and calbindin D-28k, rendering their cytosolic buffering capacity inadequate, thus making them more dependent on mitochondria to remove calcium from the cytosol than other cell types [94]. Intracellular calcium activates a number of proteins, including the protease calpain, which cleaves TDP-43 to generate aggregation-prone fragments [106], which may contribute to the TDP-43 pathology observed in 97% of ALS cases [107]. Calpain can also degrade mitofusin 2 (MFN2), a crucial mitochondrial outer membrane fusion regulator [108], and MFN2 mutations cause length-dependent, peripheral neuropathy (OMIM: 608507). This leads to impaired mitochondrial function, induction of cell death, and increased vulnerability to excitotoxicity. Wang et al. found that overexpression of MFN2 blocks the mitochondrial fragmentation caused by glutamate-induced calcium influx [105]. Blocking mitochondrial dysfunction further upstream by inhibiting calpain activation also ameliorated excitotoxicity. This suggests an important role of the protease calpain in promoting both TDP-43 pathology and mitochondrial fragmentation. Thus, an increase in intracellular calcium concentration as a result of excitotoxicity (e.g., dysregulation of AMPARs, impaired mitochondrial calcium buffering) will ultimately activate calpain and induce TDP-43 cleavage.

Chronic calcium overload of mitochondria leads to an increase in ROS production as well as oxidative stress [94]. Oxidative stress is a well-described mechanism in ALS, as biomarkers for oxidative stress are increased in ALS patients [109]. Importantly, such biomarkers can be found in (i) FALS cases linked to SOD1, (ii) familial non-SOD1 cases, such as those involving the valosin-containing protein (VCP) gene, and (iii) SALS cases involving RNA binding proteins such as TDP-43 and FUS [110]. This indicates the broad relevance of oxidative stress in ALS. Specifically, ROS can inactivate the EAAT2 glutamate transporter [62]. When the transporter is damaged, astrocytes cannot efficiently remove glutamate from the synapse, leading to glutamate accumulation [6]. Adding to this accumulation, activated microglia also produce ROS and release factors that promote the release of glutamate from astrocytes [6]. In 2004, Rao and Weiss proposed a feed-forward model for ALS progression involving mitochondrial dysfunction, suggesting that increased synaptic glutamate activates ionotropic receptors on MNs, which leads to an influx of calcium [6]. The rising intracellular calcium concentration contributes to mitochondrial dysfunction and the production of ROS, which can escape from MNs to damage the EAAT2 glutamate transporter on astrocytes. Disruption of astrocytic glutamate transport further increases the level of synaptic glutamate, causing the cycle to repeat such that glutamate will activate the neuron. This cycle emphasizes how processes happening at the synapse, in neurons, and in nearby glial cells all contribute to a feed-forward cycle of excitotoxicity.

### 3.2. ER Contribution

It is important to consider cross-talk between the mitochondria and ER, as they have an interconnected role in responding to cellular stressors. In ALS, loss of ER–mitochondria communication likely contributes to dysregulated calcium homeostasis [94]. Altogether, it is estimated that 5–20% of mitochondria are closely linked with the ER at mitochondria-associated ER membranes (MAMs). This membrane allows calcium to be exchanged between the two organelles. In mutant FUS and TDP-43 models, impaired ER–mitochondrial communication contributes to reduced uptake of calcium by mitochondria, leading to a rise in cytosolic calcium by triggering release from the ER [94]. Similarly, during ER stress, calcium can leak from the ER and be taken up by mitochondria, which disrupts mitochondrial function and promotes the generation of ROS [111].

The ER acts as a calcium sink, playing an important role in regulating cytoplasmic calcium levels [111]. Excitotoxicity disrupts the ER environment through altered calcium homeostasis, leading to ER stress and the activation of the unfolded protein response (UPR) [111] (Figure 4). There are three pathways of the UPR that ER stress can activate, each mediated by a different ER transmembrane protein: (i) inositol-requiring kinase 1 (IRE1), (ii) protein kinase RNA-activated (PKR)-like ER kinase 1 (PERK), and (iii) activating transcription factor 6 (ATF6) [112]. For IRE1-mediated UPR, in the absence of ER stress, IRE1 is inhibited by an interaction with the ER chaperone immunoglobulin binding protein (BiP). However, when misfolded proteins are present in the ER, BiP preferentially binds to these misfolded proteins, dissociating from the ER. Activation of IRE1 then leads to the cleavage of X-box binding protein 1 (XBP1), which upregulates genes involved in the UPR [113]. PERK activation causes phosphorylation of the eukaryotic translation initiation factor 2 subunit α (eIF2α), which halts most protein translation [114]. Finally, activation of ATF6 results in its transport to the cis-Golgi compartment and subsequent cleavage. Cleaved ATF6 then serves as a transcription factor to upregulate the transcription of ER stress response genes, such as XBP1. The first phases of the UPR aim to protect the cell from further ER stress by upregulating ER chaperone proteins such as BiP, protein disulfide isomerase (PDI), and others.

Proteins implicated in ALS, such as SOD1, TDP-43, and FUS, are all linked to UPR induction [112]. Cos7 cells transfected with mutant SOD1 (but not WT) exhibited SOD1 aggregates localized to the ER, along with increased ER stress [115]. Similarly, spinal cord MNs derived from mutant SOD1 Tg mice displayed an increase in ER chaperone proteins such as BiP, indicative of ER stress. Importantly, this preceded onset of motor symptoms, suggesting that SOD1-induced ER stress is upstream of motor neuron degeneration [115]. Another group uncovered an interaction between mutant SOD1 and Derlin-1, a member of the ER-associated degradation (ERAD) machinery, which induced ER stress via activation of the apoptosis signal-regulating kinase 1 (ASK1) pathway, resulting in cell death [116]. Importantly, blocking the interaction between mutant SOD1 and Derlin-1 reduced ER stress, ASK1 activation, and MN death. Supporting this finding, deletion of *ASK1* increased the lifespan of mutant SOD1 Tg mice, demonstrating that this pathway is integral to promoting the loss of MNs [116].

TDP-43 dysfunction can also be linked to ER stress. Indeed, pharmacologically induced ER stress with thapsigargin in Neuro2a cells leads to cytoplasmic TDP-43 aggregation and association with stress granules [117]. Moreover, C-terminal TDP-43 fragments colocalize with the ER and Golgi. These findings suggest that ER stress is upstream of TDP-43 pathology and that TDP-43 accumulation in the ER causes dysfunction in this organelle. Moreover, this can be directly connected to excitotoxicity, as thapsigargin causes ER stress by inhibiting Ca^2+^ ATPase [117], increasing the cytosolic calcium concentration. Thus, a feed-forward loop may arise in which rising intracellular calcium levels induce ER stress, TDP-43 cleavage, and mislocalization of TDP-43 C-terminal fragments to the ER, further disrupting ER function and intracellular calcium buffering.

Like TDP-43, mutant FUS exhibits nuclear clearance and cytoplasmic aggregation in ALS [118]. Using MN-like mouse NSC-34 cells, redistribution of mutant FUS to the cytoplasm was shown to trigger ER stress [118]. More specifically, mutant FUS was found to colocalize with PDI, an ER chaperone protein protective against ER stress. In this context, PDI may be protective by preventing the misfolding of FUS. This study suggests that FUS pathology lies upstream of ER stress, unlike TDP-43 [118], although each can initiate the same feed-forward mechanism in which aberrant protein cleavage or misfolding induces ER stress, exacerbating protein aggregation and further ER stress.

In summary, there is compelling evidence that both FALS and SALS are linked to ER stress, which may result from increased intracellular calcium levels downstream of excitotoxicity. Indeed, kainic acid (KA), which activates KA glutamate receptors, was found to induce excitotoxic cell death in rat hippocampal neurons by activating the UPR and inducing eIF2α phosphorylation (inhibiting global translation) [119]. Intriguingly, treatment with salubrinal, which inhibits eIF2α dephosphorylation (preserving translation inhibition), significantly decreased both KA-induced ER stress and neuronal death in vivo (male Wistar rats) and in vitro (rat hippocampal neurons) [119]. This study is important for several reasons. First, it provides evidence that excitotoxicity induced by activation of glutamate receptors triggers ER stress. Second, it demonstrates that inhibition of ER stress by salubrinal is protective against excitotoxicity, suggesting that ER stress is a major component of excitotoxicity-induced cell death.

In ALS, we described evidence for extensive cross-talk not only between the ER and mitochondria, but also amongst different players in the excitotoxicity cascade, as interactions between neurons and glia, processes at the synapse, and calcium-buffering organelles are all highly connected. Considering the various primary and secondary pathways described here, therapeutic targets aimed at reducing excitotoxicity may be protective (at least partially) in a broad population of ALS patients.

## 4. Conclusions and Therapeutic Potential

**Figure 5:**
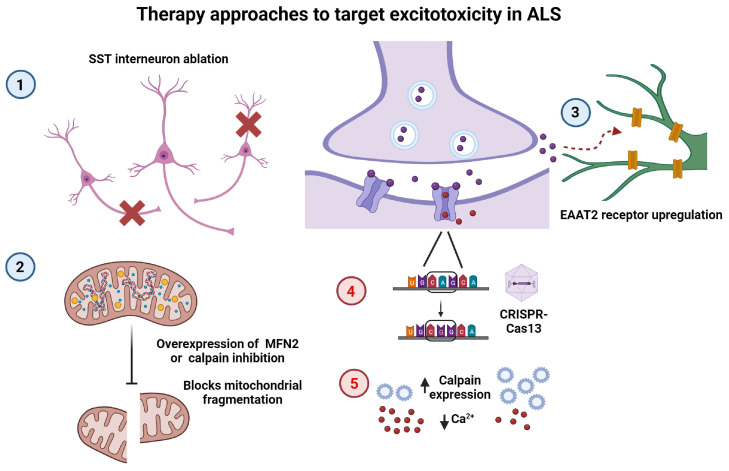
Existing and potential therapeutic strategies to target excitotoxicity in ALS. Examples of therapy interventions, previously proposed (blue circles, 1–3) or untested (red circles, 4–5), to target the excitotoxicity pathway: (1) SST interneuron ablation (red cross) restores normal excitability of layer-5 pyramidal neurons [91]. (2) Overexpression of MFN2 or inhibition of calpain activation blocks calcium-induced mitochondrial fragmentation [108]. (3) Upregulation of astrocytic EAAT2 receptors reduces excess glutamate at the synapse [135]. (4) AAV delivery of CRISPR-Cas13 machinery to replace ADAR2 function in editing the GluR2 pre-mRNA. (5) Upregulation of calpains or other neuronal calcium-buffering proteins to decrease cytosolic Ca^2+^ concentration.

In this review, we discussed the origin and effects of excitotoxicity in ALS, first by characterizing primary contributions to excitotoxicity, defined as processes occurring at the synapse. This included (i) upregulation of calcium-permeable AMPA receptors, such as those that lack the GluR2 subunit or those that contain the unedited GluR2 subunit; (ii) dysfunction of the EAAT2 astrocytic glutamate transporter, leading to reduced clearance of this neurotransmitter from the synapse; (iii) increased release of glutamate from the presynaptic neuron due to increased calcium in the terminal; and (iv) the contribution of inhibitory interneurons to MN hyperexcitability. We then discussed secondary pathways, in which increased intracellular calcium resulting from influx through calcium-permeable receptors, such as AMPARs or NMDARs, causes (i) changes to mitochondrial morphology and function; (ii) production of ROS, which can damage EAAT2 transporters; (iii) ER stress and the induction of the unfolded protein response; and (iv) aberrant cross-talk between the mitochondria and ER, all of which may lead to neuronal death. Given the number of cellular processes involved in excitotoxicity, there are many opportunities for therapeutic intervention in FALS and SALS. As noted earlier, there is already evidence for several interventions in this pathway. For example, overexpression of MFN2 or inhibition of calpain activation can block calcium-induced mitochondrial fragmentation, ameliorating excitotoxicity in vivo [108]. We also noted that normal excitability is restored in layer-5 pyramidal neurons when SST interneurons are ablated [91] (Figure 5).

In addition to these experimentally tested targets, we offer several novel suggestions. Since increased calcium influx is at the very root of excitotoxicity, we propose limiting the entry of Ca^2+^ by designing therapies to compensate for reduced GluR2 editing due to ADAR2 loss of function. Perhaps AAV vectors delivering CRISPR-Cas9/13 for genome editing of the GluR2 pre-mRNA in the CNS could be used as a technique to ensure that the GluR2 subunit is impermeable to calcium. In addition, given that MNs are particularly susceptible to excitotoxicity because they have low expression of calcium-buffering proteins, upregulation of calcium-buffering or calcium-activated proteins (such as calpains) could also be protective.

While there is a great deal of research detailing excitotoxicity in ALS, there are still important questions that remain to be answered. We can clearly describe how excitotoxicity affects individual neurons, namely through increased activation of glutamate receptors, allowing calcium to build up to deleterious concentrations within the cell, but it seems less clear how excitotoxicity spreads from one neuron to another. Perhaps excitotoxic neurons release factors into the extracellular space and propagate disease by inducing excitotoxicity in surrounding neurons. These factors may include cytoplasmic inclusions like TDP-43 or even extra glutamate itself [120]. Alternatively, we described how ROS can escape from neurons and affect nearby astrocytes, potentially propagating an excitotoxic response.

Mouse models often fall short of recapitulating human physiology. It has been postulated that ALS is a human-specific disorder originating in the neocortex [121]. Evidence for this is based on certain aspects unique to humans in the cortico-motoneuronal (CM) system. Direct connections exist between specific cortical projection cells and their bulbar and spinal targets, the α-motoneurons. These have direct control of bulbar and spinal cord activity through monosynaptic CM connections [122]. The CM system serves special functions, including adaptive, complex motor behaviors that are largely limited to non-human primates and humans (e.g., split-hand, split-leg syndromes) [123]. In primates, corticospinal neurons exhibit several significant features distinguishing them from mice. These include short-duration spikes [124], some fibers with fast conduction velocities [125], and widespread expression of the fast K+ channels [126]. Eisen et al. (2017) suggested that these primate-specific features may make these neurons particularly vulnerable to TDP-43 pathology [121]. We suggest that this may be one of the reasons that ALS mice, even TDP-43 transgenic mice, do not exhibit all pathological features of human ALS [127]. In fact, one TDP-43 overexpression mouse model displays ubiquitin-positive aggregates, MN loss, and early death, but these aggregates do not contain TDP-43 [128]. Several studies remind us to be cautious in our use of animal models in research. For example, the h-current (Ih), a slow, depolarizing current activated by hyper-polarization [129,130], more strongly affects the membrane properties of supra-granular pyramidal neurons in the human temporal cortex compared to neurons in the temporal association area of mice. In agreement with this, membrane properties of the human neurons affected by h-currents are more sensitive to pharmacological blockade compared to mice.

Properties of voltage-gated channels like h-channels affect how neurons respond to stimulation. The potential for mouse and human neurons to spike differently emphasizes the need to investigate this topic further. Interestingly, the human endplate, which is the area of the skeletal muscle on which the motor neuron synapses, is one of the smallest of vertebrate endplates and has a low quantal content, meaning less neurotransmitter (ACh) is released onto the skeletal muscle for each action potential [131,132]. As a form of compensation, human skeletal muscle has greater infolding, such that the increased surface area holds more voltage-gated sodium channels, allowing efficient propagation [133]. However, these properties make human NMJs more vulnerable to changes in neurotransmitter release than mouse NMJs. Even a small decrease in the amount of neurotransmitter released from the motor neuron can substantially affect neuromuscular transmission. In contrast, mice can safely tolerate marked decreases in neurotransmitter release up to 50% [131,133,134]. In sum, these findings demonstrate that human NMJs have a lower “safety factor” [132], which means that experiments in mice aiming to mimic human motor neuron pathologies at the NMJ must be carefully designed to take physiological differences into account.

Furthermore, a recurring issue in many neurological diseases is that late detection precludes any form of intervention because by the time the disease is detected, substantial loss of neurons has already occurred, and function cannot be restored. Therefore, there is a pressing need for research on biomarkers of ALS. Hyperexcitable neurons could serve as a biomarker, which is already achieved to a small extent using electromyography. However, use of this technique could be more widespread to enhance detection of changes in cortical excitability before considerable loss of neurons occurs.

Excitotoxicity has been proposed as an important disease mechanism in several neurodegenerative disorders through both cell-autonomous and non-cell-autonomous processes. While some features of excitotoxicity have been reported across multiple diseases, the specific population of neurons affected appears to be context-dependent. Thus, further study of excitotoxic processes across multiple model systems has the potential to inform our broad understanding of age-associated neurodegeneration, while also providing avenues for specific disease-modifying therapy development.
